# Natural Killer Cells Generated From Human Induced Pluripotent Stem Cells Mature to CD56^bright^CD16^+^NKp80^+/-^ *In-Vitro* and Express KIR2DL2/DL3 and KIR3DL1

**DOI:** 10.3389/fimmu.2021.640672

**Published:** 2021-05-04

**Authors:** Johanna Euchner, Jasmin Sprissler, Toni Cathomen, Daniel Fürst, Hubert Schrezenmeier, Klaus-Michael Debatin, Klaus Schwarz, Kerstin Felgentreff

**Affiliations:** ^1^ Institute for Transfusion Medicine, Ulm University, Ulm, Germany; ^2^ International Graduate School in Molecular Medicine, Ulm University, Ulm, Germany; ^3^ Department of Pediatrics and Adolescent Medicine, Ulm University Medical Center, Ulm, Germany; ^4^ Institute for Transfusion Medicine and Gene Therapy, Medical Center – University of Freiburg, Freiburg, Germany; ^5^ Faculty of Medicine, University of Freiburg, Freiburg, Germany; ^6^ Institute for Clinical Transfusion Medicine and Immunogenetics Ulm, German Red Cross Blood Service Baden-Württemberg-Hessen, Ulm, Germany

**Keywords:** induced pluripotent stem cells (iPSC), natural killer (NK) cells, hematopoietic progenitor cells (HPC), NK cell differentiation, OP9-DL1

## Abstract

The differentiation of human induced pluripotent stem cells (hiPSCs) into T and natural killer (NK) lymphocytes opens novel possibilities for developmental studies of immune cells and *in-vitro* generation of cell therapy products. In particular, iPSC-derived NK cells gained interest in adoptive anti-cancer immunotherapies, since they enable generation of homogenous populations of NK cells with and without genetic engineering that can be grown at clinical scale. However, the phenotype of *in-vitro* generated NK cells is not well characterized. NK cells derive in the bone marrow and mature in secondary lymphoid tissues through distinct stages from CD56^bright^CD16^-^ to CD56^dim^CD16^+^ NK cells that represents the most abandoned population in peripheral blood. In this study, we efficiently generated CD56^+^CD16^+^CD3^-^ NK lymphocytes from hiPSC and characterized NK-cell development by surface expression of NK-lineage markers. Hematopoietic priming of hiPSC resulted in 31.9% to 57.4% CD34^+^CD45^+^ hematopoietic progenitor cells (HPC) that did not require enrichment for NK lymphocyte propagation. HPC were further differentiated into NK cells on OP9-DL1 feeder cells resulting in high purity of CD56^bright^CD16^-^ and CD56^bright^CD16^+^ NK cells. The output of generated NK cells increased up to 40% when OP9-DL1 feeder cells were inactivated with mitomycine C. CD7 expression could be detected from the first week of differentiation indicating priming towards the lymphoid lineage. CD56^bright^CD16^-/+^ NK cells expressed high levels of DNAM-1, CD69, natural killer cell receptors NKG2A and NKG2D, and natural cytotoxicity receptors NKp46, NKp44, NKp30. Expression of NKp80 on 40% of NK cells, and a perforin^+^ and granzyme B^+^ phenotype confirmed differentiation up to stage 4b. Killer cell immunoglobulin-like receptor KIR2DL2/DL3 and KIR3DL1 were found on up to 3 and 10% of mature NK cells, respectively. NK cells were functional in terms of cytotoxicity, degranulation and antibody-dependent cell-mediated cytotoxicity.

## Introduction

Natural Killer (NK) cells are an important part of the innate immune system. They play a crucial role in the defense of viral infections and cancer containment, as well as in immune regulation (1).

In recent years, NK cells became favorable candidates for cellular anti-cancer immunotherapies because of their HLA-independent cytotoxic capacity against malignant and virally infected cells (2). The option to generate therapeutic NK cells from human induced pluripotent stem cells (hiPSC) opens new possibilities for potential genetic engineering and adoptive transfer. Although chimeric antigen receptor (CAR) expression was mostly studied in T cells, CAR constructs can also improve NK cell-mediated killing in hiPSC-derived NK cells (3). In contrast to T cells, NK cells do not depend on specific receptors to recognize their targets and do not cause graft versus host disease (GvHD) (4). Besides engineering and expansion for immunotherapies, iPSCs provide a platform for NK-cell differentiation studies and human disease modeling for which patient-specific gene variations can be introduced.

Several groups established protocols for the differentiation of iPSC- and embryonic stem cell (ESC)-derived NK cells after induction of hematopoietic progenitor cells (HPCs) (5–13). In order to differentiate iPSCs to CD34^+^CD45^+^ HPCs, embryonic bodies (EB) are generated using different approaches, such as spinning of single cell iPSCs in round-shaped wells (spin EBs), culture on murine stroma cells, or direct induction of iPSC monolayer fragments in media with cytokines inducing differentiation towards the hematopoietic lineage. HPCs are often enriched by cell sorting or cell separation of CD34^+^ and/or CD45^+^ cells, and subsequently placed on murine feeder cells (e.g. AFT024, OP9, MS-5, EL08-1D2) in medium containing IL-3 (during the first week), IL-7, IL-15, SCF, IL-2, and Flt3L. NK-cell differentiation without usage of xenogeneic stromal feeder cells has also been reported (6). However, limited information is available about efficiency of these protocols and the phenotype of generated NK cells.

Human NK cells derive from a common lymphoid progenitor cell in the bone marrow and their maturation can be categorized into 5 major stages (14). The last step of differentiation from CD56^bright^CD16^-^ into CD56^dim^CD16^+^ NK cells takes place in secondary lymphoid tissues (SLTs), whereas CD56^bright^CD16^+^ NK cells are considered as functional intermediates (15). CD56^bright^ NK cells express CCR7, CXCR3 and L-selectin (CD62L) that allow homing to SLTs, liver, skin, and uterus where they interact with MHC molecules, antigens and homeostatic cytokines (16). CD56^dim^ NK cells are characterized by a different profile of chemokine receptors and represent the most abandoned population in peripheral blood, but can also be found in bone marrow, lung, spleen and breast tissue (17). Although they are generally considered as precursors to CD56^dim^ NK cells, little is known about the maturation process and egress of CD56^bright^ NK cells from SLTs. Mature NK cells characteristically express inhibiting and activating receptors that bind to MHC class I molecules and regulate inflammatory and cytotoxic function. The family of KIRs (killer cell immunoglobulin-like receptors) (18) consists of several receptor types containing two or three domains with long (e.g. KIR2DL1 and KIR3DL1) and short cytoplasmic tails (e.g. KIR2DS1, KIR2DS2), whereas receptors with long tails are known to transmit inhibitory and those with short tails activating signals. Transmembrane NKG2 receptors (NKG2A, B, C, E, H) pair with the CD94 molecule to heterodimeric surface receptors, whereas NKG2D forms homodimeric receptors. While CD94/NKG2A and NKG2B transmit inhibitory signals, all the others are activating receptors. Activating cytotoxicity receptors (NCRs), NKp46 (NCR1, CD335), NKp44 (NCR2, CD336), NKp30 (NCR3, CD337), contain immunoglobulin-like domains binding to tumor antigens, viral proteins, and some bacterial components (19). CD56^bright^CD16^-^ and CD56^dim^CD16^+^ NK cells show significant differences in their expression profile of these surface markers that is acquired during NK cell education. CD56^bright^CD16^-^ NK cells can be further divided into stage 4a and 4b by expression of NKp80 (20). Whereas NKG2D and NCRs are expressed on both subsets, CD94/NKG2A is mainly found on CD56^bright^CD16^-^ cells, and the expression of KIRs and LIRs (leukocyte immunoglobulin-like receptors) is more specific to CD56^dim^CD16^+^ (21).

IPSC-derived NK cells generated *in-vitro* are supposed to lack self MHC interactions, although the impact of xenogeneic feeder cells on the maturation processes is not well understood.

In this study, we aimed to establish an efficient protocol to generate mature and functional NK cells from hiPSC with optimized efficiency. Furthermore, we characterized the phenotype of generated NK cells in terms of maturation and activation status by NK-lineage marker expression, as well as functional studies of degranulation and cytotoxicity.

## Material and Methods

### Cell Lines, Cell Culture, and Characterization of iPSCs

The mouse stroma cell line OP9-DL1 (gift from J. Plum laboratory, Gent, Belgium) was used as feeder for differentiating NK cells. The human K562 cell line (DMSZ, ACC Nr. 10) and the murine mastocytoma FcR^+^ cell line P815 (gift from Prof. Dr. S. Ehl, University of Freiburg, Freiburg, Germany) were used for functional NK cell studies. A human healthy control iPSC line derived from reprogramming of newborn human foreskin fibroblasts (NuFF) C.3 using the CytoTune iPS Sendai Reprogramming kit (ThermoFisher). Karyotype analysis showed no chromosomal abnormalities (**Supplementary Figure 1A**). Pluripotency-associated proteins SSEA4, OCT4, TRA-1-60 and NANOG were analyzed by immunofluorescent staining and flow cytometry (**Supplementary Figure 1B**). Expression of pluripotency- (*DNMT3B, GDF, hTERT, NANOG, OCT4*, *SOX)* and reprogramming-associated genes (*MYCC*, *KLF4)* was assessed in a quantitative PCR normalized on NHDF fibroblasts (**Supplementary Figure 1C**). IPSCs were differentiated into all three germ layers *in-vitro*, which was confirmed by expression of lineage specific markers analyzed by flow cytometry (endoderm: SOX17^+^/CD184^+^, mesoderm: CD140b^+^, CD144^+^, ectoderm: SOX2^+^/PAX6^+^ (**Supplementary Figure 1D**). HLA and KIR genotyping was performed on genomic DNA (Wizard^®^ genomic DNA purification kit, Promega) by SSP-PCR (22). Material and methods for cell culture and characterization of iPSCs are described in detail in the supplementary material.

### *In-Vitro* Differentiation of Human iPSCs to CD34^+^/CD45^+^ Hematopoietic Progenitor Cells

IPSCs were placed in one well of a 6-well culture plate coated with Vitronectin XF™ (STEMCELL Technologies) and grown to 60% - 100% confluency. With the help of the StemPro™ EZPassage™ Disposable Stem Cell Passaging Tool (ThermoFisher), colonies were cut into squares, carefully washed and transferred into one well of a 6-well ultra-low attachment plate. During overnight incubation, cell clusters developed into EBs of different sizes. To induce differentiation towards CD34^+^/CD45^+^ HPCs, the STEMdiff™ Hematopoietic Kit (STEMCELL Technologies) was used according to manufacturer’s instructions. EBs from cell suspension were collected using a 40µm cell strainer (Falcon) and rinsed off with 1ml differentiation medium A (1ml/12 well). 1% Penicillin-Streptomycin (ThermoFisher) was added to the differentiation medium A and B. On day 12, the suspension cells were harvested and passed through a 40μm cell strainer. No positive selection of HPCs was performed prior NK-cell differentiation.

### *In-Vitro* Differentiation of Hematopoietic Progenitor Cells to NK Cells

On day -1, 5 and 12 of NK-cell differentiation, 2x10^5^ OP9-DL1 feeder cells/well were prepared in 6-well culture plates. On day 0, 2.5x10^5^ HPCs (CD34^+^/CD45^+^) were plated on prepared feeder cells in 2ml NK differentiation medium [56.6% DMEM high glucose (ThermoFisher), 28.3% Ham’s F-12 Nutrient Mix (ThermoFisher), 15% heat-inactivated human AB serum (Valley Biomedical), 2mM L-glutamine (ThermoFisher), 25μM β-mercaptoethanol (ThermoFisher), 5ng/ml Sodium selenite (Sigma-Aldrich), 50μM Ethanolamine (Sigma-Aldrich), 20mg/L L-ascorbic acid (Sigma-Aldrich), 1% Penicillin-Streptomycin; 20ng/ml recombinant human stem cell factor (SCF), 20ng/ml recombinant human IL-7, 10ng/ml recombinant human IL-15, 10ng/ml recombinant human Flt3 ligand, and 5ng/ml recombinant human IL-3 (PeproTech)] (7, 23). All cytokines were added from stock solutions on the day of use; recombinant human IL-3 was used only during the first week. Half medium changes were performed on day 3, 6, 9, 13 and 16 with NK-cell medium containing doubled cytokine concentrations. On day 6 and 13, progenitor cells were replaced on feeder cells. On day 6 (week 1 - w1), 13 (week 2 - w2) and 20 (week 3 – w3), cells were analyzed for NK-cell marker expression by flow cytometry. In order to remove OP-DL1 stroma cells, NK cells were filtered through a 40µm strainer. Of note, contamination with OP9-DL1 could be excluded by gating on the much smaller NK cell population that was further selected by expression of hematopoietic and lymphoid lineage marker. The protocol was further optimized by treatment of OP9-DL1 feeder cells with 10mg/µl mitomycin C (MMC) (STEMCELL Technologies) for 2h in order to prevent proliferation. After treatment, cells were rinsed 3x with phosphate buffer saline (PBS).

### Flow Cytometry

2x10^4^ - 2.5x10^5^ cells were washed twice with PBS and stained with antibody mixes in 100µl PBS or staining buffer (PBS, 1% FCS) for 15 to 30 min at room temperature (RT) in the dark. The following antibodies and isotype controls were used at concentrations of 1:100: Anti-Human CD43-PE (STEMCELL Technologies), Anti-Human CD45-FITC (STEMCELL Technologies), APC anti-human CD34 (BioLegend), PE anti-human CD3 (BioLegend), FITC anti-human CD16 (BioLegend), APC anti-human CD56 (NCAM) (BioLegend), FITC anti-human CD94 (BioLegend), PE anti-human CD117 (c-kit) (BioLegend), FITC anti-human CD161 (BioLegend), PE anti-human CD335 (NKp46) (BioLegend), PE anti-human CD7 (BioLegend), PE anti-human CD244 (2B4) (BioLegend), PE anti-human CD57 (BioLegend), PE anti-human CD226 (DNAM-1) (Miltenyi Biotec), PE anti-human CD159c (NKG2C) (Miltenyi Biotec), PE anti-human CD159a (NKG2A) (Miltenyi Biotec), PE anti-human NKp80 (Miltenyi Biotec), APC anti-human CD62L (BioLegend), PerCP/Cyanine5.5 anti-human CD56 (NCAM) (BioLegend), APC/Cyanine7 anti-human CD45 (BD Biosciences), APC anti-human CD69 (Miltenyi Biotec), APC anti-human CD181 (CXCR1) (Miltenyi Biotec), APC anti-human CD336 NKp40 (Miltenyi Biotec), APC anti-human CD337 (NKp30) (Miltenyi Biotec), APC anti-human CD314 (NKG2D), (Miltenyi Biotec), PE anti-human CD158b (KIR2DL2/L3, NKAT2) (BioLegend), APC anti-human CD158e1 (Kir3DL1, NKB1) (BioLegend), APC Mouse IgG1, κ Isotype Ctrl (BioLegend), FITC mouse IgG1, κ Isotype Ctrl (BioLegend), PE Mouse IgG1, κ Isotype Ctrl (BioLegend). After two washes, cells were suspended in 50-100µl PBS or staining buffer and analyzed on a BD Accuri C6 Flow Cytometer (Becton Dickinson Biosciences), or a BD FACSAria™ Cell Sorter (BD Biosciences), respectively.

### Cytotoxicity and Degranulation Studies

NK cells generated on OP9-DL1 cells were harvested after w3, and incubated with K562 cells at effector:target (E:T) ratios of 1.5:1, 3:1, 6:1, 12:1, 25:1, 50:1, and 100:1 at 37°C for 1h. K562 cells were labeled using CellTrace™ far red cell proliferation kit (ThermoFisher) according to manufacturer’s instructions. Lysis of K562 cells was calculated based on the same numbers of K562 cells cultured without NK cells. For degranulation studies, CD107a (PE anti-human CD107a; BioLegend) was analyzed on surfaces of non-permeabilized NK cells cultured with K562 at the same E:T ratios used for the cytotoxicity assay. Additionally, NK cells were fixed and permeabilized (PermWash, BD Biosciences), and subsequently stained for perforin (PE anti-Perforin, human, mouse, rat; Miltenyi Biotec), granzyme B (PE anti-Granzyme B, human, mouse, rat; Miltenyi Biotec), and interferon-gamma (APC anti-human IFNγ, BioLegend). IFNγ production was further analyzed in NK cells stimulated with phorbol-12-myristat-13-acetat (PMA) (Sigma-Aldrich) (10ng/ml) and Calcium Ionophore A23187 (Sigma-Aldrich) (1µg/ml) at 37°C for 3h, followed by BD GolgiPlug™ (BD Biosciences) (2µl/ml) for 2h. Cells were analyzed on a BD FACSAria™ Cell Sorter.

### Antibody-Dependent Cell-Mediated Cytotoxicity

The redirected antibody-dependent cell-mediated cytotoxicity (ADCC) was evaluated based on a protocol reported by Byceson et al. (24). The murine FcR^+^ cell line P815 was coated with mouse anti-human CD16 antibody (BioLegend) or IgG1 kappa isotype control (BioLegend). Coated and uncoated P815 were subsequently used as target cell line for NK cells in various E:T ratios (1.5:1, 3:1, 6:1, 12:1, 25:1, 50:1, and 100:1). P815 were labeled using CellTrace™ (ThermoFisher) according to manufacturer’s instructions, and percentages of lysed P815 cells were analyzed. In addition, CD107a surface expression was studied on NK cells in response to P815 as mentioned above.

### Statistical Analysis

All experiments were performed 3-5 times. GraphPad Prism 5 software was used for statistical analysis; *p*-values on multiple comparisons were calculated using two-way ANOVA and bonferroni post test; *p*<0.05 was considered significant. Mean values and standard errors of the mean (SEM) are plotted. NK cell differentiation efficiencies per HPC were calculated using Microsoft Excel.

## Results

### Human iPSC-Derived NK Cells Can Be Generated on OP9-DL1 Feeder Cells

**Figure 1:**
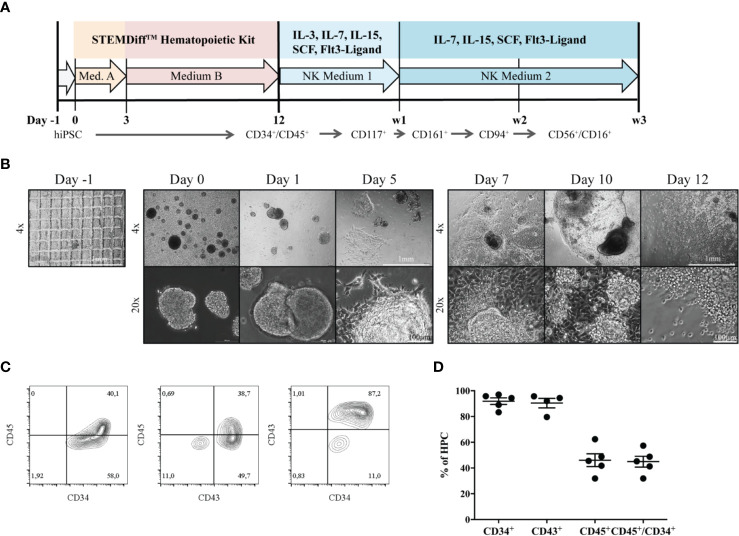
Generation of HPCs from hiPSCs. **(A)** An outline of the protocol is shown. During the first 12 days, HPCs were generated from hiPSC using the StemDiff™ Hematopoietic kit, and were subsequently differentiated to mature NK cells on OP9-DL1 feeder cells over three weeks. **(B)** The morphology of EBs and emerging HPCs during 12 days of differentiation is shown. On day -1 a confluent iPSC layer has been cut into uniform squares. Day 0: EBs of different sizes occur. Day 3 and 5: EBs start to enlarge and attach to the culture vessel surface. Subsequently, attached EBs continue to spread forming large clumps. From day 7, progenitor cells appear as bright and round cells budding off from the attached structures. The bar represents 1mm and 100µm, corresponding to 4x and 20x magnification, respectively. **(C)** Representative flow cytometric analysis of CD34, CD43 and CD45 expression on day 12. **(D)** Percentages of CD34^+^, CD43^+^, CD45^+^ and CD45^+^CD34^+^ cells obtained from multiple independent flow cytometric experiments on day 12 of HPC differentiation (n = 5, CD43: n=4). Bars represent mean and standard deviation.

Differentiation of iPSCs to HPCs was induced as described before (25) (**Figure 1A**). During the process of differentiation, EBs attached to the culture surface to build a monolayered structure (**Figure 1B**). At day 7, the first progenitors emerged as small, bright cells budding off from attached EB formations. HPCs in suspension were used for NK-cell differentiation on day 12, after purity was assessed by expression of surface markers CD34, CD45 and CD43 (**Figure 1C**). In five independent experiments, 83.3% - 97% of HPCs expressed CD34, and 31.9% - 57.4% were CD34^+^CD45^+^ double positive. CD43, a common marker to measure hematopoietic induction efficiency besides CD34 (26), could be detected on the vast majority of 79.6% - 95.7% HPCs (**Figure 1D**).

**Figure 2:**
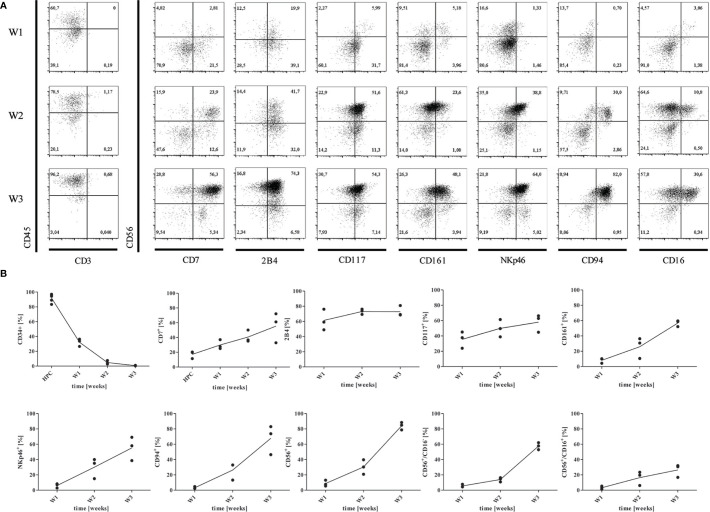
Differentiation of HPCs into NK cells. **(A)** Representative flow cytometry analysis of CD7, CD244 (2B4), CD117, CD161, CD335 (NKp46), CD94, CD16, and CD56 expression gated on CD45^+^CD3^-^ cells obtained from co-culture with OP9-DL1 at w1, w2, and w3, respectively. Gating strategy and isotype controls are shown in **Supplementary Figure 3**. **(B)** Mean expression levels of CD34, CD7, 2B4, CD117, CD161, NKp46, CD94, CD56, CD56^+^CD16^-^, and CD56^+^CD16^+^ at w1, w2, and w3 (n=3).

Enrichment of CD34^+^CD45^+^ progenitors was not needed and NK cells were differentiated over 3 weeks on OP9-DL1 feeder cells based on previous reports (7, 23). We compared inactivation of OP9-DL1 feeder with MMC to non-inactivated feeder cells, which significantly impacted on the yield of mature NK cells (**Supplementary Figure 2A**), but not on maturation or functional qualities. During the differentiation process, developing NK cells became denser and smaller in size (**Supplementary Figure 2B**), and changed their expression of surface markers (**Figures 2A, B**). Cells were assessed weekly for expression of HPC- and NK lineage-markers CD34, CD7, 2B4, CD117, CD161, CD94, CD335, CD56, and CD16 by flow cytometry (**Figures 2A, B**). Undifferentiated iPSCs were used as negative control to set up gating strategies (**Supplementary Figure 3**).

The hematopoietic lineage marker CD45 could be detected on 44,5% of progenitors in week 1 (w1), and on 94,9% of cells obtained in week 3 (w3) (**Figure 2A**), whereas the mean expression of CD34 strongly declined from w1-w3 in 5 independent experiments. All cells were CD3^-^ at any time point. CD7 was expressed by a mean of 15.7% HPCs, and 28%-56% NK progenitors at w1 and w2/w3 indicating commitment to the lymphoid lineage (27). From w2 and w3, the majority of CD7^+^ cells co-expressed CD56. CD117 (c-kit) expression could increasingly be detected on NK progenitors from w1-w3 up to 66%. According to the model of NK-cell differentiation suggested by Freud et al. (14), NK progenitors express either CD117 or CD94, whereas CD117 can be detected on early CD34^+^CD117^+^CD94^-^ progenitors. However, CD94 was found on NK progenitors from w2 at high levels (**Figure 2A**). Defining another marker of NK cell lineage commitment, CD161 was accessed on up to 69% of NK progenitors from w2 and w3. CD335 (NK-p46), a natural cytotoxicity receptor (NCR) highly specific for NK cells, could be constantly detected from w2. We further investigated expression of the immunoregulatory transmembrane receptor 2B4 (CD244) that is expressed on all NK cells mediating both activating and inhibiting signals through engagement with its ligand CD48 (28). In our study, 2B4 appeared on 49%-81% of NK progenitor cells and mature NK cells from w1-w3. CD56^+^ cells could be detected from w2, and constituted over 85% of cells at the endpoint. Accordingly, CD56^bright^CD16^-^ shifted to CD56^bright^CD16^+^ cells from w2 to w3 accounting for an average of 58.7% and 27% generated cells, respectively. Of note, all cells expressing CD94 or NKp46 were also CD56^+^ (**Figure 2A**). It has been described that CD56^bright^/CD94^+^ NK cells further develop to CD56^dim^/CD94^+^ and finally to CD56^dim^/CD94^-^ NK cells (29). However, iPSC-derived NK cells did not mature to CD56^dim^CD16^+^ NK cells in our studies.

**Figure 3:**
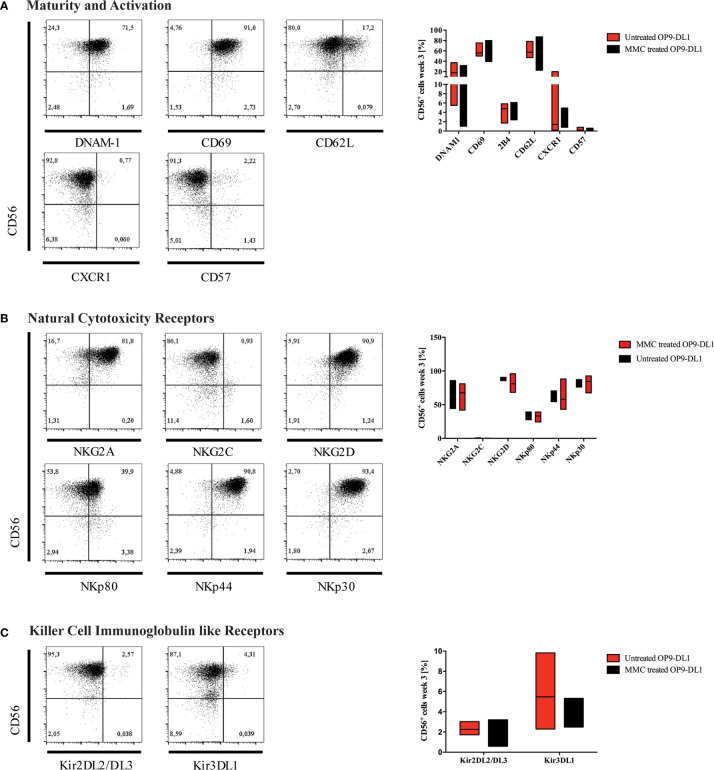
Characterization of generated NK cells for expression of maturity markers and NK-cell receptors. Representative flow cytometry analyses for the expression of **(A)** maturity markers DNAM-1, CD69, CD62L, CXCR1, and CD57; **(B)** natural cytotoxicity receptors (NCR), and **(C)** killer cell immunoglobulin-like receptors (KIR) are shown on CD45^+^CD3^-^CD56^+^ NK cells obtained at w3. On the right-hand side, expression levels of investigated surface markers gated on CD45^+^CD3^-^CD56^+^ NK cells are summarized in whisker plots **(A–C)** (n=3), and compared to NK cells obtained from culture on OP9-DL1 feeder cells treated with MMC.

On average, 2.4 CD45+CD3-CD56+ NK cells developed per applied CD34^+^/CD45^+^ HPC progenitor after three weeks of differentiation when plated on non-inactivated OP9-DL1 feeder cells. However, inactivation of OP9-DL1 resulted in higher efficiency with a mean of 8.6 NK cells obtained per applied CD34^+^/CD45^+^ progenitor (**Supplementary Figure 2A**), but did not impact on maturation (**Figure 3**). Presumably, MMC treatment inhibits further proliferation of OP9-DL1 feeder cells and reduces their consumption of media nutrients.

### Human iPSC-Derived NK Cells Mature to CD56^bright^CD16^+^ KIR2DL2/L3^+^KIR3DL1^+^ NK Cells *In-Vitro*

After three weeks of differentiation, NK cells were characterized for expression of maturity and activation markers, NCRs, and KIRs by flow cytometry (**Figures 3A–C**). The majority of generated NK cells expressed DNAM-1 (CD226), which functions as an adhesion molecule that synergizes with activating receptors and mediates cytotoxicity upon interaction with its ligand CD155 and CD112. Whereas DNAM-1 is unanimous on NK progenitors, it is downregulated as NK cells mature, and DNAM-1^+^ and DNAM-1^-^ NK cells can be found in the periphery. CD69 was highly expressed on most CD56^+^ NK cells indicating an activated phenotype. In contrast, the chemokine receptor CXCR1, which is mostly found on mature CD56^dim^CD16^+^ NK cells, and the senescence marker CD57 were absent (**Figure 3A**). CD62L could be detected on up to 17% of cells.

CD94/NKG2 is a family of C-type lectin receptors which are expressed predominantly on the surface of NK cells and a subset of CD8^+^ T cells. They recognize non-classical MHC class I glycoproteins and stimulate or inhibit cytotoxic activity of NK cells. Expression of both activating (NKG2D) and inhibiting NK cell receptors (NKG2A) was detected, however, expression of NKG2C was much lower (**Figure 3B**). NCRs play a crucial role in NK-mediated recognition and killing of virus-infected or tumor cells that express NCR-ligands. Due to interaction of their signaling cascades, NCRs are collectively present at either high or low surface densities (30).

The NCRs NKp46 (NCR1, CD335), NKp44 (NCR2, CD336), and NKp30 (NCR3, CD337) were expressed in high levels on most NK cells (**Figure 3B**). Of note, NKp80 [killer cell lectin-like subfamily F, member 1 (KLRF1)], that has been defined to discriminate stage 4a and 4b NK cells (20), was detected on up to 40% of iPSC-derived NK cells.

KIRs are expressed at late stages of NK cell differentiation and their repertoires are shaped during NK cell education (31). Development of mature NK cells expressing KIR receptors requires cellular interaction with HLA-antigens. It has been reported in previous studies that NK cells can acquire expression of certain KIRs such as KIR2DL3 through interaction with stroma cells *in-vitro* (32), although it is generally accepted that iPSC-derived NK cells mostly show a KIR negative phenotype (33). In our study, however, KIR2DL2/L3 and KIR3DL1 were both detected on up to 3% and 10% of CD56^+^ NK cells, respectively (**Figure 3C**). KIR genotyping of the iPSC line used for NK-cell differentiation confirmed the presence of *2DL2*, *2DL3* and *3DL1* genes (**Supplementary Table 1**). In congruence with our results, low frequencies of KIR expression on iPSC-derived NK cells has recently been reported in other studies (34, 35).

MMC treatment of OP9-DL1 feeder cells did not impact on the phenotype of generated NK cells.

### IPSC-Derived NK Cells Are Functional for Degranulation, Cytotoxicity and ADCC

**Figure 4:**
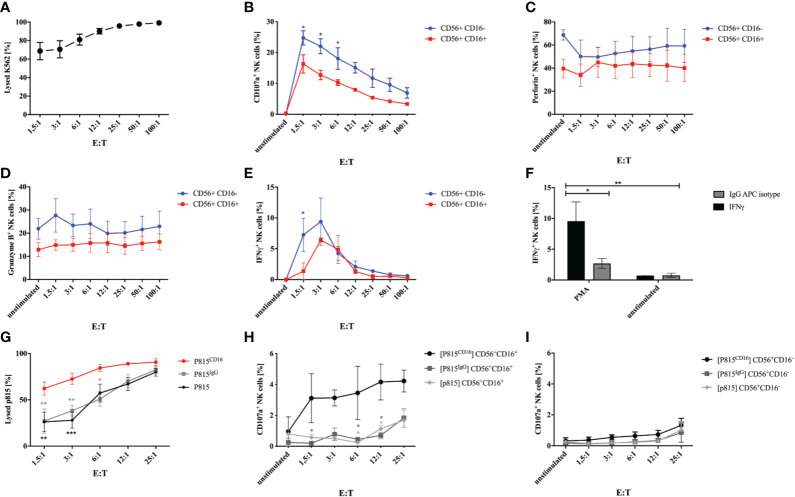
Degranulation and cytotoxicity studies on generated NK cells. Functional studies were performed on NK cells obtained after 3 weeks of differentiation. **(A)** NK cells were incubated with K562 cells labeled with a cell tracer far red dye (CTFR) at indicated E:T ratios for 1h. Percentages of lysed K562 are shown. Expression of CD107a **(B)**, perforin **(C)**, granzyme B **(D)**, and IFNγ was studied on CD56^+^CD16^+^ and CD56^+^CD16^-^ NK cells that were cultured with K562 cells at indicated E:T ratios. Basal expression of unstimulated NK cells without K562 co-culture are shown as controls. **(F)** Expression of IFNγ was analyzed in NK cells in response to stimulation with PMA/Calcium Ionophore and compared to non-stimulated cells and isotype staining. **(G)** ADCC of NK cells was investigated in response to P815 cells either uncoated or coated with murine anti-CD16 antibody or IgG isotype. NK cells were incubated with P815 at indicated E:T ratios. Percentages of lysed P815 are shown. In addition, CD107a expression was assessed on CD56^+^CD16^+^ **(H)** and CD56^+^CD16^-^ NK cells **(I)** following culture with P815 under the same conditions as for investigation of the ADCC. Shown are mean values and SEM of 3 experiments (n=3). Statistics were calculated using two-way ANOVA and Bonferroni post test (* p ≤ 0,05, ** p ≤ 0,01, *** p ≤ 0,001). In some cases error bars were smaller than the symbol and could therefore not been shown. E:T, effector:target ratio.

In order to test whether generated NK cells obtained at w3 are functional, we evaluated degranulation and cytotoxicity when co-cultured with K562 tumor cells. Incubation of NK cells with K562 cells in E:T ratios of 1.5:1 to 100:1 for 1h resulted in efficient killing of target cells (**Figure 4A**). In addition, NK cells expressed increasing amounts of CD107a in response to culture with K562 (**Figure 4B**). Interestingly, CD107a was expressed on up to 29% of CD56^+^CD16^-^ NK cells in contrast to 21% on CD56^+^CD16^+^ cells plated at highest E:T ratios. Perforin and granzyme B was predominantly expressed on CD56^+^CD16^-^ NK cells, which declined in response to K562 co-culture (**Figures 4C, D**). IFNγ production was strongly induced by K562 cells, although particularly in CD56^+^CD16^-^ NK cells and only at lower E:T ratios up to 12:1 (**Figure 4E**). Stimulation with PMA and Calcium Ionophore resulted in a strong IFNγ response compared to non-stimulated cells and isotype staining (**Figure 4F**). In order to investigate the antibody-dependent cell-mediated cytotoxicity, we co-cultured iPSC-derived NK cells with murine FcR^+^ P815 coated with a murine anti-human CD16 antibody or an isotype control, respectively. E:T- depended cytotoxicity could already be observed in response to unlabeled and IgG-labeled P815 cells, however, cytotoxicity was significantly increased against CD16-coated P815 (**Figure 4G**). Of note, P815^CD16^-dependent CD107a surface expression could only be observed in CD56^+^CD16^+^ NK cells and not in response to uncoated or isotype-coated P815 (**Figures 4H, I**).

## Discussion

Generation of NK cells from hiPSCs has been described by several groups within the last decades using different approaches that result in various efficiencies (1, 6, 8, 11–13, 33, 36). We report a robust and highly efficient protocol to generate NK cells from hiPSCs, which we characterized in detail for maturation status and function. We compared the phenotypes of NK cells derived on OP9-DL1 feeders with and without anti-proliferative treatment with MMC. Although differentiation on inactivated feeders resulted in higher yields of NK cells obtained, no difference in expression of maturity markers and specific NK-cell receptors was observed. On average, 8.6 mature NK cells could be obtained per HPC used for NK cell propagation on inactivated OP9-DL1 feeder cells.

Several stroma cell lines have been used for NK cell differentiation (AFT024, M2-10B4, OP9) from hiPSC (37–41). OP9-DL1, a bone-marrow derived stroma cell line that expresses the Notch ligand Delta-like 1, has been reported for differentiation of human CD34^+^ progenitors (14) and peripheral blood derived iPSC. Although mandatory for the induction of T cell development (42), it is known that Notch-1 signaling contributes to efficient development of NK cells in humans (43, 44). Of note, neither CD3^+^ T, nor CD3^+^CD56^+^ natural killer T cells were detected in our studies.

Human NK cells derive from HPCs in the bone marrow (45, 46). However, several models exist that discuss linear development starting from a common lymphoid progenitor (CLP) versus a branched model, in which NK cells potentially derive from multiple precursors, including common myeloid progenitors (CMP) (47). A recent study describes the development of extra-embryonic or yolk sac derived cytotoxic NK cells emerging from erythro-myeloid progenitors (48) that could also be observed in hiPSC-derived NK cells. Menon and colleagues proposed an *in vitro* differentiation process from HPCs to NK lineage committed cells (CD117^+^ and CD122^+^), NK precursor cells (CD161^+^ and CD56^+^), immature NK cells (CD94^+^, CD335^+^ and CD56^+^), and eventually mature NK cells (CD45^+^, CD335^+^, CD56^+^, CD158b^+^ and CD16^+^) (12). A similar pathway has been described by Freud et al. that divides human NK cell maturation of secondary lymphoid tissues into five different stages (49). The majority (around 90%) of peripheral blood NK cells is CD56^dim^CD16^+^. In contrast, CD56^bright^CD16^-^ NK cells comprise 5-15% of peripheral blood NK cells, and reside predominantly in secondary lymphoid organs. From this stage, a linear terminal maturation is proposed to the CD56^dim^ stage *in vivo* (14, 45). The latter produce high amounts of cytolytic granules, perforin and granzyme B (50, 51), and express CXCR1 at higher density compared to CD56^bright^CD16^-^ cells (52). CD56^dim^ NK cells further differentiate by losing NKG2A expression and acquiring KIR receptors and CD57. In recent years, NK cell education has been studied in more detail. Maturation of NK cells depends on cellular interaction with HLA-antigens that shapes the repertoire of KIR expression (18). Inhibitory KIRs are characterized by long cytoplasmic tails (KIR-L), and activating KIRs have short cytoplasmic tails (KIR-S). In the process of education, the strength of the inhibitory signal dictates the efficiency of the NK cell effector function. Although *in-vitro* derived NK cells are generally considered to be KIR negative, development of inhibitory KIRs, KIR2DL2/L3 in particular, was demonstrated after co-culture with feeder cells, such as OP9-DL1 and mesenchymal stem cells (MSCs) (32, 33).

IPSC-derived CD56^bright^CD16^-/+^ NK cells generated with our protocol displayed a highly activated phenotype shown by upregulation of CD69, and CD62L. However, DNAM-1 (CD226), could be detected on only one third of NK cells in our study. DNAM-1 is expressed early in NK cell development and associated with education (53). It is constantly expressed on NK progenitors, but can be downregulated on mature NK cells. 2B4 could be detected on up to 30% of NK cells, including immature CD56^-^ progenitors from w1 and w2. 2B4 is expressed on CLPs and NK progenitors (54), but can be dynamically regulated in peripheral blood, in response to viral infections (55).

In our study, NK-cell receptors such as NKG2A, NKG2D, NKp30, NKp44, and NKp46 were highly expressed, but senescence markers CXCR1 and CD57 were negative. Expression of NKp80 on up to 40% of CD56^bright^ NK cells characterizes their maturation up to stage 4b (14). Accordingly, perforin and granzyme B were detected at high to moderate levels, associated with efficient degranulation and cytotoxicity capacities in response to K562 target cells. Of note, we observed CD107a surface expression, and cytosolic expression of perforin, granzyme B and IFNγ predominantly in CD56^+^CD16^-^ NK cells compared to CD56^+^CD16^+^ cells. We were able to show ADCC against CD16-coated P815 cells and P815^CD16^-mediated CD107a expression of CD56^+^CD16^+^ NK cells. Cytotoxic function and tumor surveillance have recently been studied extensively in iPSC-derived NK cells (35), which we could confirm for NK cells generated with our protocol.

Stage 4a NK cells are generally expected as KIR^-^ (54). As reported before for NK cells generated on feeder cells (32–35), we could detect up to 3% of KIR2DL2/DL3 on NK cells obtained at w3. Interestingly, we confirmed also expression of KIR3DL1 on 2.3-9.8% of mature NK cells in three independent experiments. KIR3DL1 interacts with HLA-B molecules with the Bw4 serological motif, whereas KIR2DL2/DL3 is specific to HLA-C C1 (18). KIR genotyping of iPSC revealed a positive result for all three KIR genes suggesting that also the HLA type of the donor cell line could impact on NK cell repertoire. Genetic and epigenetic modulations on somatic cells that served as source for iPSC, as well as interactions of developing NK cells with differentiating iPSC and hematopoietic progenitors could impact on KIR repertoire in addition to feeder cells. Although NK cell education *in-vitro* and the impact of stem cell sources need to be evaluated further, these results demonstrate that CD56^bright^ NK cells are able to acquire KIR expression to some extent.

## Conclusion

We report a robust protocol for *in-vitro* generation of human NK cells that could be used for cellular therapies or modeling of human immunodeficiencies. CD3^-^CD56^bright^CD16^+/-^ NK cells can be differentiated from hiPSC up to stage 4b (NKp80^+^) on OP9-DL1 stroma cells and are highly functional in terms of degranulation, cytokine production and cytotoxicity including ADCC. IPSC-derived CD56^bright^ NK cells can acquire KIR expression, although in a limited fashion and most likely dependent on donor source. NK cell yield can be considerably increased through inactivation of feeder cells with MMC without impacting on maturation or functional properties.

## Data Availability Statement

The original contributions presented in the study are included in the article/**Supplementary Material**. Further inquiries can be directed to the corresponding author.

## Author Contributions

KF and KS conceptualized, planned and supervised the study. JE and JS performed the experiments and analyzed the data. TC provided the iPSC line. DF performed the HLA and KIR genotyping of the iPSC line. HS and K-MD supervised the study. JE and KF wrote the manuscript, which was approved by JS, TC, HS, K-MD, and KS. All authors contributed to the article and approved the submitted version.

## Funding

JE and JS are fellows of the International Graduate School in Molecular Medicine, Ulm. KF holds a grant from the German Research Foundation (FE1253/3-1).

## Conflict of Interest

The authors declare that the research was conducted in the absence of any commercial or financial relationships that could be construed as a potential conflict of interest.
