# Supplementary material for: Natural Killer Cells Generated From Human Induced Pluripotent Stem Cells Mature to CD56brightCD16+NKp80+/- In-Vitro and Express KIR2DL2/DL3 and KIR3DL1
Source: Front Immunol. 2021 May 4;12:640672. doi: 10.3389/fimmu.2021.640672 (PMC8129508; doi:10.3389/fimmu.2021.640672)
Supplement: Supplementary file 1 [file DataSheet_1.docx]

**Supplementary Material and Methods**

1. **Supplementary Methods**
   1. **Cell culture**

Culture medium for stroma cells contained 90% MEM alpha (ThermoFisher), 10% of heat-inactivated, sterile filtered fetal calf serum (FCS) (Biowest) and 1% Pen/Strep (ThermoFisher).

IPSCs were maintained on hESC-qualified Corning® Matrigel® matrix in mTeSR1^TM^ medium (STEMCELL Technologies) supplemented with 1% Pen/Strep, or on Vitronectin XF^TM^ (STEMCELL Technologies) in StemMACS^TM^ iPSC Brew XF media (Miltenyi Biotec), respectively. ReLeSR^TM^ (STEMCELL Technologies) was used for detaching cells at a confluence of 60% - 100% according to the manufacturer’s instructions. Depending on density, iPSCs were seeded 1:10 to 1:50 into matrix-coated culture dishes and 10µM Rock-inhibitor (Y-27632 dihydrochloride; Sigma-Aldrich) was added to the culture medium.

- 1. **Karyotype analysis**

The karyotype was analyzed on iPSCs grown on two 10cm dishes to confluency of 50% - 70%. 93.2µl/10 cm^2^ of a 20mg/l colchicine stock solution (Eurobio) was added to the medium and cells were incubated for 2h at 37°C. After two washing steps, iPSCs were detached using Accutase^®^ (Sigma-Aldrich) solution and analyzed at the Institute of Human Genetics, University Hospital Ulm.

- 1. **Immunofluorescent staining**

Cells were fixed for 20min with Roti^®^-Histofix 4% (Carl Roth) at RT followed by permeabilization with 0.2% Triton-X (Sigma-Aldrich) in PBS^+/+^ for 30min at RT, and blocking in 5% goat serum (Sigma-Aldrich)/ PBS^+/+^ for 2h at RT. The primary antibodies anti-SSEA4 (Abcam) (1:50), anti-TRA-1-60 (R) (Abcam) (1:100), anti-OCT4 (Abcam) (1:100), anti-NANOG (Abcam) (1:50) were diluted in blocking solution. Cells were incubated for 2h at RT or in the dark at 4°C overnight. The secondary antibodies goat-anti-rabbit IgG (H&L) (Abcam), and goat anti-mouse IgG, IgM (H+L) (ThermoFisher) were diluted 1:1000 in blocking solution and cells were incubated for 30min at 37°C in the dark. Subsequently, cells were fixed again with Roti^®^-Histofix 4% for 10min at 4°C. The cell’s nuclei were counterstained with 2μg/ml 4′,6-Diamidino-2-phenylindole dihydrochloride (Sigma-Aldrich) in PBS^+/+^ for 3min at RT, protected from light. Cells were imaged on a confocal microscope using a 10x objective.

- 1. **Flow Cytometry**

For pluripotency studies, 5x10^5^ iPSC were incubated in single cell suspension with anti-SSEA4-PerCP Vio700 (Miltenyi Biotec) (1:10) and Alexa Flura®488 anti-human Tra-1-60-R (BioLegend) (1:20) in staining buffer 30min at RT in the dark. After permeabilization using the cell signaling buffer kit A (Miltenyi Biotec) according to manufacturer’s instructions, iPSCs were stained with anti-Nanog-PE (Miltenyi Biotec) and anti-Oct3/4-APC (Milenyi Biotec) (1:10) for 30min at RT in the dark. The following isotype controls were used: REA control antibody (I), human IgG1, APC (Miltenyi Biotec), REA control antibody (I), human IgG1, PE (Miltenyi Biotec), Cells were analyzed on a FACSAria^TM^ Cell Sorter.

**1.5 Quantitative real time PCR**

RNA was isolated using the RNeasy Plus Mini Kit (Qiagen) and QIAshredder spin columns (Qiagen), and cDNA was synthesized using the SuperScript^TM^ II Reverse Transcriptase (ThermoFisher) according to the manufacturer’s protocol. The Rotor-Gene SYBR^®^ Green PCR Kit (Qiagen) was used to perform qRT-PCR according to manufacturer’s instructions. RNA of three reference genes *HPRT1* (Hs_HRPT1_1_SG_QuantiTect Primer Assay; Qiagen)*, RPL13A* (F: 5’-CGGACCGTGCGAGG-3’, R: 5’-CACCATCCGCTTTT-3’), and *UBC* (F: 5’-CTAGTTCCGTCGCAGCCGGGA-3’, R: 5’-TGGTGTCACTGGGCTCAACCTCG-3’), six pluripotency associated genes *DBMT3B, GDF3, hTERT, NANOG, OCT4, SOX2*, and two reprogramming associated genes *KLF4, MYCC* were amplified (sequences reported before) (1, 2). A 10µl reaction was set up using 10ng of template. Three technical and three biological replicates per sample were analyzed on a Rotor-Gene Q device. The initial denaturation was performed at 95°C for 5min, followed by 45 cycles of denaturation (95°C for 10sec) and annealing/elongation (60°C for 20sec). Relative expression ratios (R) were calculated according to Pfaffl (3).

**1.6 Differentiation of iPSC into three germ layers**

Differentiation potential into lineages of all three germ layers was confirmed using the StemMACS^TM^ trilineage differentiation kit (Miltenyi Biotec) according to manufacturer’s instructions. Expression of CD144 (anti-CD144-FITC, human; Miltenyi Biotec), CD140b (Anti-CD140b-APC, human; Miltenyi Biotec), SOX17 (anti-SOX17-PE, human; Miltenyi Biotec), CXCR4 (anti-CD184 (CXCR4)-APC, human; Miltenyi Biotec), SOX2 (anti-SOX2-FITC, human; Miltenyi Biotec), and PAX6 (anti-PAX6-PE, human; Miltenyi Biotec) was analyzed by flow cytometry. NHDF fibroblast cells served as negative control in all assays.

1. **Supplementary Tables**

| **HLA genotype** | | | | | | | | |
| --- | --- | --- | --- | --- | --- | --- | --- | --- |
| *HLA class I* | | A*02:01:01G, *32:01:01G | | | | | | |
|  |  | B*52:01:01G, *57:01:01G | | | | | | |
|  |  | C*06:02:01G, *12:02:01G | | | | | | |
| *HLA class II* | | DRB1*07:01:01G, *15:02:01G | | | | | | |
|  |  | DQB1*03:03:02G, *06:01:01G | | | | | | |
|  |  | DPB1*04:01:01G | | | | | | |
| **KIR genotype** | | | | | | | | |
| 2DL1 | 2DL2 | | 2DL3 | 2DL4 | 2DL5 | 2DS1 | 2DS2 | 2DS3 |
| positive | positive | | positive | positive | negative | negative | positive | negative |
| 2DS4 | 2DS5 | | 2DP1 | 3DL1 | 3DL2 | 3DL3 | 3DS1 | 3DP1 |
| positive | negative | | positive | positive | positive | positive | negative | positive |

**Supplementary Table 1: HLA and KIR genotype of healthy control iPSC**

1. **Supplementary Figures**

3.1 Supplementary Figure 1: Karyotype and pluripotency analysis of hiPSC

(A) Karyogram of hiPSCs. (B) Immunofluorescence staining for pluripotency markers SSEA4, OCT4, TRA-1-60 and NANOG (scale bar 250µm). NHDF fibroblasts served as negative control (not shown). (C) Quantitative real-time PCR showing the expression of pluripotency associated genes *DNMT3B, GDF, hTERT, NANOG, OCT4* and *SOX*. Reprogramming-associated genes *MYCC* and *KLF* were used as controls. Results were normalized on NHDF fibroblasts (dashed line). (D) Flow cytometry analysis of SSEA4, TRA1-60, NANOG, and OCT3/4. (E) IPSCs (a) were differentiated into mesodermal (b), endodermal (c), ectodermal (d) lineages (scale bar 100µm), which was confirmed by expression of CD144 and CD140b (mesoderm), SOX17/CXCR4 (endoderm), SOX2/PAX6 (ectoderm) by flow cytometry. Percentages of cells expressing the indicated markers are shown in the bar chart.

**3.2 Supplementary Figure 2: NK cell yield obtained from inactivated and non-inactivated feeder cells and morphology of NK cell progenitors**

(A) Yield of mature CD45^+^CD3^-^CD56^+^ NK cells after week 1-3 developed from HPC plated on OP9-DL1 feeder cells. Mean and variation of yields obtained from 3 independent experiments are shown. Two Way ANOVA, Bonferroni´s post test, *** p<0,001. (B) Morphology of NK cell progenitors on OP-9DL1 feeder cells from week 1 to week 3 (scale bar 100µm).

**3.3 Supplementary Figure 3: Gating strategy of NK cell markers assessed by flow cytometry**

The gating strategy of NK-lineage markers expressed during different stages of differentiation is shown for iPSCs and NK cells obtained on w3. Cells were gated on CD45^+^CD3^-^, and indicated markers are shown against CD56. Isotypes were used as negative controls and gates were set accordingly. Expression of indicated markers on NK-cell progenitors is shown in **Figure 2A** and **Figure 3**.

1. **Supplementary References**

1. Felgentreff K, Du L, Weinacht KG, Dobbs K, Bartish M, Giliani S, et al. Differential role of nonhomologous end joining factors in the generation, DNA damage response, and myeloid differentiation of human induced pluripotent stem cells. *Proc Natl Acad Sci U S A* (2014) 111(24):8889-8894.

2. Park IH, Zhao R, West JA, Yabuuchi A, Huo H, Ince TA, et al. Reprogramming of human somatic cells to pluripotency with defined factors. *Nature* (2008) 451(7175):141-146.

3. Pfaffl MW A new mathematical model for relative quantification in real-time RT-PCR. *Nucleic Acids Res* (2001) 29(9):e45.
