# Supplementary figures and images for: Natural Killer Cells Generated From Human Induced Pluripotent Stem Cells Mature to CD56brightCD16+NKp80+/- In-Vitro and Express KIR2DL2/DL3 and KIR3DL1
Source: Front Immunol. 2021 May 4;12:640672. doi: 10.3389/fimmu.2021.640672 (PMC8129508; doi:10.3389/fimmu.2021.640672)

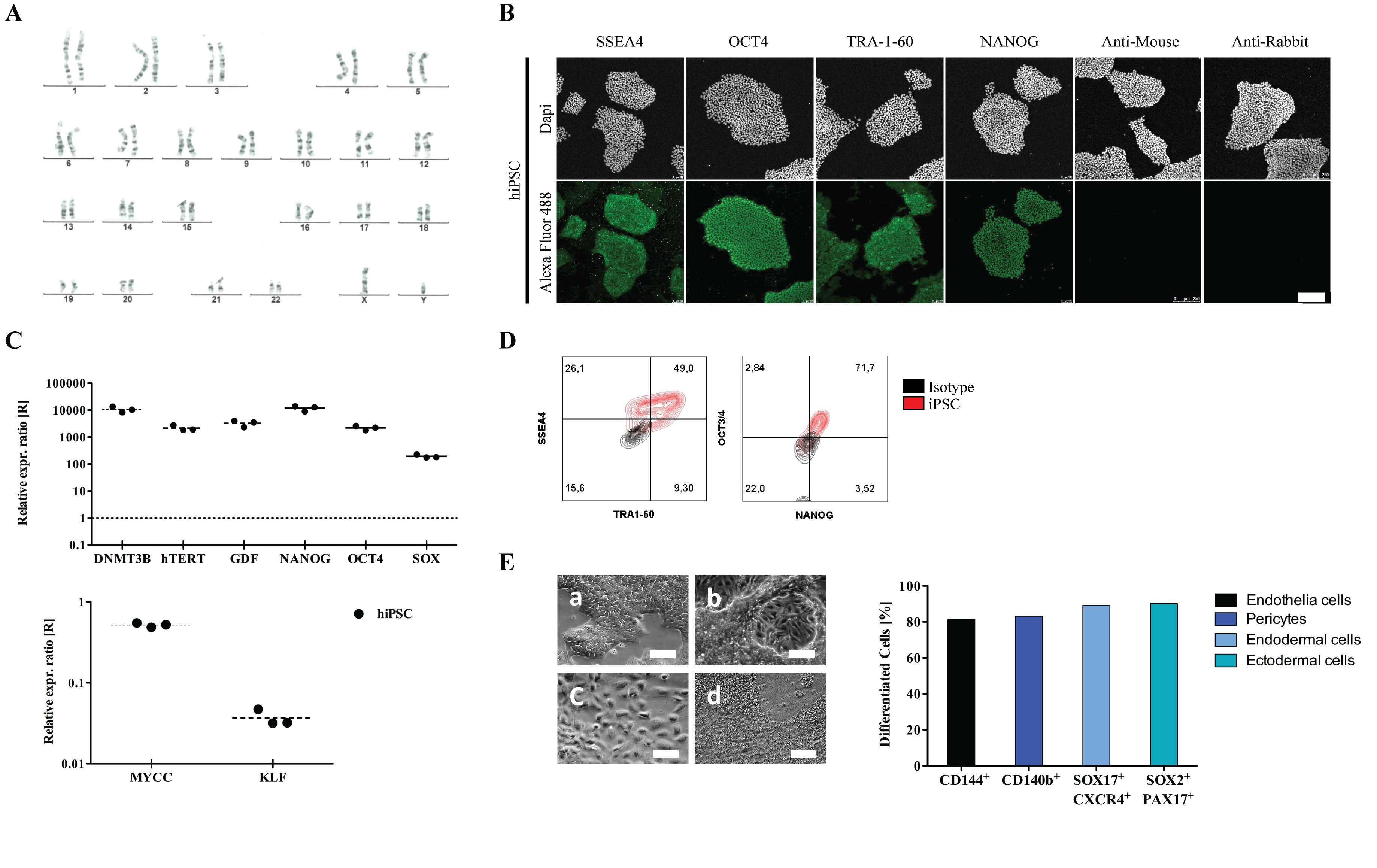

Supplement: Supplementary file 2 [file Image_1.tif]

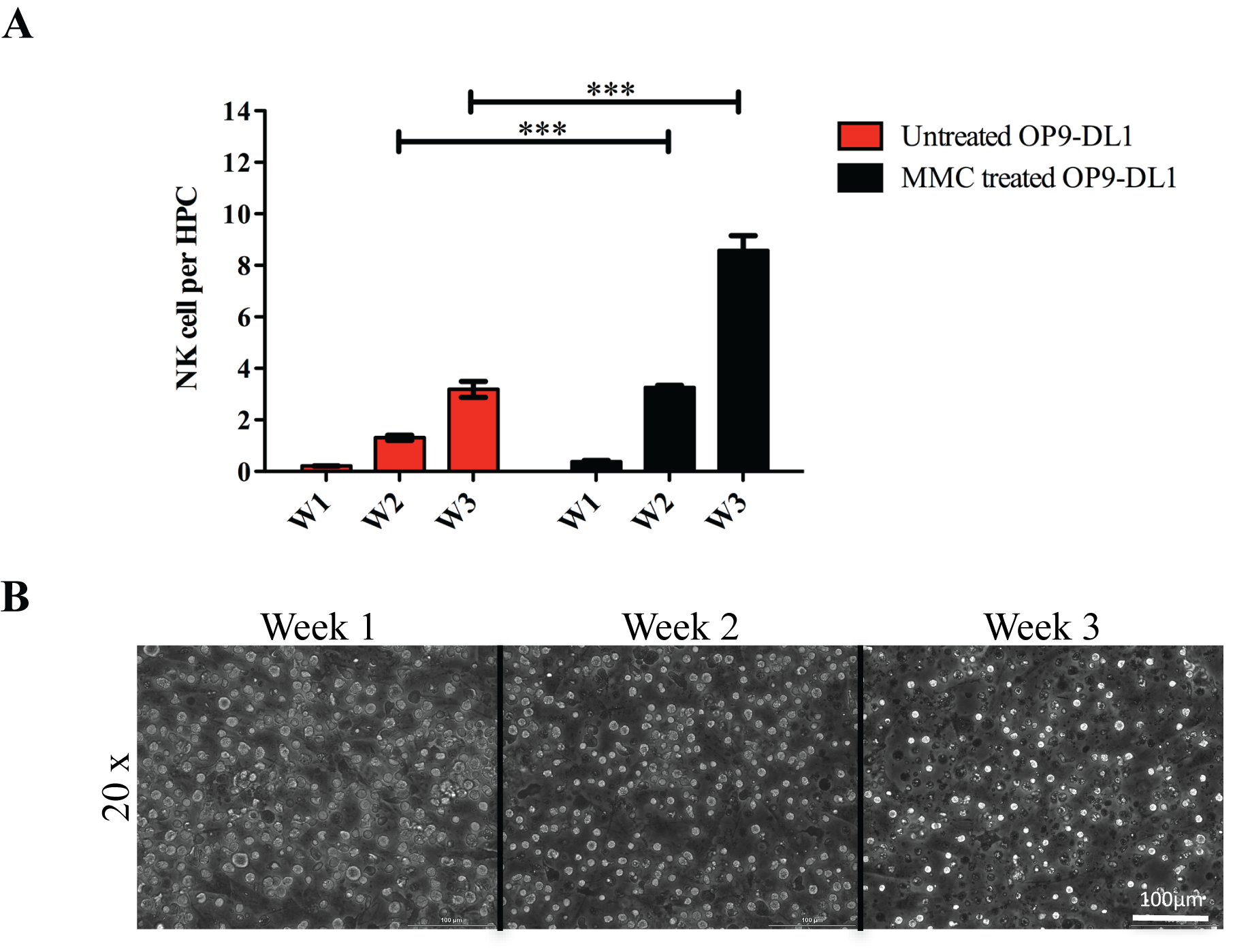

Supplement: Supplementary file 3 [file Image_2.tif]

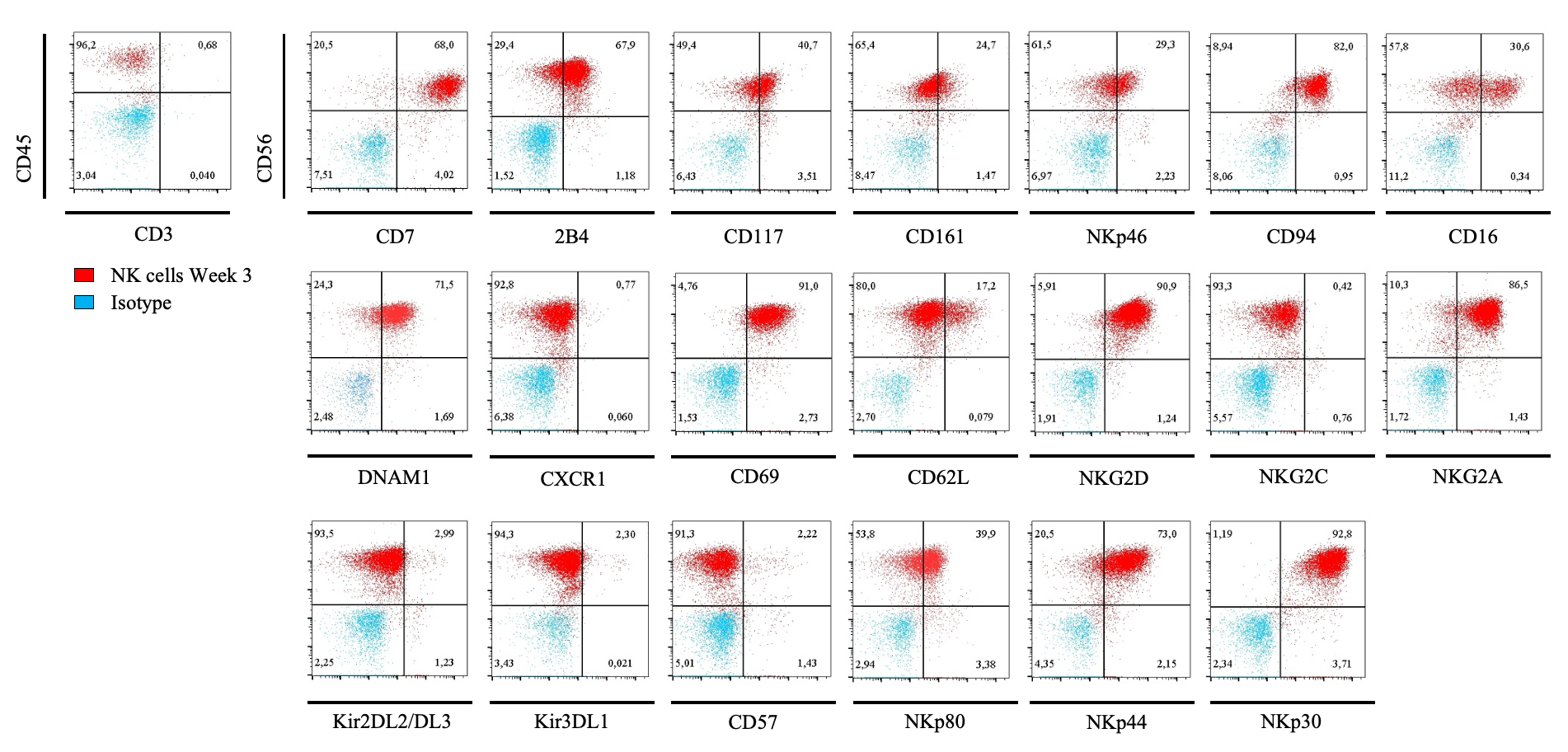

Supplement: Supplementary file 4 [file Image_3.tiff]
